# Quorum Sensing as a Target for Controlling Surface Associated Motility and Biofilm Formation in *Acinetobacter baumannii* ATCC^®^ 17978^TM^

**DOI:** 10.3389/fmicb.2020.565548

**Published:** 2020-09-30

**Authors:** Celia Mayer, Andrea Muras, Ana Parga, Manuel Romero, Soraya Rumbo-Feal, Margarita Poza, José Ramos-Vivas, Ana Otero

**Affiliations:** ^1^Departamento de Microbioloxía e Parasitoloxía, Facultade de Bioloxía, Edificio CIBUS, Universidade de Santiago de Compostela, Santiago de Compostela, Spain; ^2^National Biofilms Innovation Centre, Biodiscovery Institute and School of Life Sciences, University of Nottingham, Nottingham, United Kingdom; ^3^Microbioloxía, Instituto de Investigación Biomédica da Coruña, Centro de Investigacións Científicas Avanzadas da Coruña, Universidade da Coruña, A Coruña, Spain; ^4^Servicio de Microbiología, Hospital Universitario Marqués de Valdecilla–Instituto de Investigación Valdecilla, Santander, Spain

**Keywords:** *Acinetobacter baumannii*, quorum sensing, surface-associated motility, biofilm, quorum quenching, extracellular DNA

## Abstract

The important nosocomial pathogen *Acinetobacter baumannii* presents a quorum sensing (QS) system (*abaI*/*abaR*) mediated by acyl-homoserine-lactones (AHLs) and several quorum quenching (QQ) enzymes. However, the roles of this complex network in the control of the expression of important virulence-related phenotypes such as surface-associated motility and biofilm formation is not clear. Therefore, the effect of the mutation of the AHL synthase AbaI, and the exogenous addition of the QQ enzyme Aii20J on surface-associated motility and biofilm formation by *A. baumannii* ATCC^®^ 17978^TM^ was studied in detail. The effect of the enzyme on biofilm formation by several multidrug-resistant *A. baumannii* clinical isolates differing in their motility pattern was also tested. We provide evidence that a functional QS system is required for surface-associated motility and robust biofilm formation in *A. baumannii* ATCC^®^ 17978^TM^. Important differences were found with the well-studied strain *A. nosocomialis* M2 regarding the relevance of the QS system depending on environmental conditions The *in vitro* biofilm-formation capacity of *A. baumannii* clinical strains was highly variable and was not related to the antibiotic resistance or surface-associated motility profiles. A high variability was also found in the sensitivity of the clinical strains to the action of the QQ enzyme, revealing important differences in virulence regulation between *A. baumannii* isolates and confirming that studies restricted to a single strain are not representative for the development of novel antimicrobial strategies. Extracellular DNA emerges as a key component of the extracellular matrix in *A. baumannii* biofilms since the combined action of the QQ enzyme Aii20J and DNase reduced biofilm formation in all tested strains. Results demonstrate that QQ strategies in combination with other enzymatic treatments such as DNase could represent an alternative approach for the prevention of *A. baumannii* colonization and survival on surfaces and the prevention and treatment of infections caused by this pathogen.

## Introduction

The genus *Acinetobacter* comprises Gram-negative, strictly aerobic coccobacilli that are widely present in the environment but also includes a variety of species that cause opportunistic nosocomial infections like septicemia, pneumonia, endocarditis, meningitis, skin, wound, and urinary tract infections (Towner, 2009). *Acinetobacter baumannii*, the most relevant pathogenic species in the genus, has emerged as one of the most troublesome hospital-acquired pathogens because the increase in the prevalence of multidrug-resistant (MDR) strains has reduced the treatment options for this pathogen (Peleg et al., 2008; Roca et al., 2012; Lee et al., 2017; Moubareck and Halat, 2020). Therefore, a better understanding of the mechanisms controlling the expression of virulence traits and propagation in *Acinetobacter* spp. has become critical for the discovery and development of new therapeutic strategies.

In *Acinetobacter* spp. a complex network of sensors controls the expression of virulence factors integrating intra- and extracellular signals (Harding et al., 2018; Wood et al., 2018a; De Silva and Kumar, 2019). Among these, quorum sensing (QS), a mechanism of control of gene expression dependent on bacterial cell density, seems to play a central role, constituting an interesting target for the development on antimicrobial strategies. *Acinetobacter* spp. present a QS system homologous to the canonical LuxI/LuxR system of Gram-negatives that produces and sense QS signals belonging to the family of the acyl-homoserine-lactones (AHLs) that is known as AbaI/AbaR (Niu et al., 2008; Bhargava et al., 2010; Mayer et al., 2018). The system was first identified in *A. nosocomialis* M2, formerly classified as *A. baumannii* (Carruthers et al., 2013) and later found in *A. baumannii* (Smith et al., 2007; Niu et al., 2008; Mayer et al., 2018) and other species of the genus of both human or environmental origin (Kang and Park, 2010; Bitrian et al., 2012; How et al., 2015; Oh and Choi, 2015). *N*-hydroxydodecanoyl-L-homoserine lactone (OHC12-HSL) is the main QS signal produced by the clinically relevant species belonging to the *A. calcoaceticus*–*A. baumannii* complex, including the well-studied strains *A. nosocomialis* M2 and *A. baumannii* ATCC^®^ 17978^TM^, although other AHLs are also produced in smaller amounts (Niu et al., 2008; Chan et al., 2011, 2014; Clemmer et al., 2011; How et al., 2015; Mayer et al., 2018). *In vitro*, these signals are produced only in static cultures (Mayer et al., 2018), indicating a strong correlation between QS and biofilm formation and/or surface attachment. Besides producing QS signals, the capacity to enzymatically degrade these molecules, a process known as Quorum Quenching (QQ), has been described in several *Acinetobacter* spp. of environmental and clinical origin (Kang et al., 2004; Chan et al., 2011; Kim et al., 2014; Ochiai et al., 2014; Arivett et al., 2015; López et al., 2017; Mayer et al., 2018). Up to 8 putative QQ enzymes have been found in the genome of *A. baumannii* ATCC^®^ 17978^TM^ and, in this strain, QQ activity correlates with the disappearance of the AHLs from the culture media, indicating that AHL production may be self-regulated (Mayer et al., 2018).

QS controls many different functions in *Acinetobacter* spp., including some key virulence factors such as motility, biofilm formation and other stress responses (Gaddy and Actis, 2009; Roca et al., 2012; Eze et al., 2018; Harding et al., 2018; Mayer et al., 2020; Shin et al., 2020), although most work was done with *A. nosocomialis* M2 (Niu et al., 2008; Clemmer et al., 2011; Stacy et al., 2012; Oh and Choi, 2015). Surface-associated motility is related to virulence since it has been identified as a common trait in clinical isolates of *A. baumannii* (Eijkelkamp et al., 2011) and increased motility has been related to increased adherence and lethality in *Caenorhabditis elegans* assays (Eijkelkamp et al., 2013). Several regulatory proteins have been described as involved in the control of surface-associated motility in *Acinetobacter* spp. (Mayer et al., 2018; Moubareck and Halat, 2020). The AHL-mediated QS system seems to be central in this regulatory network since an impairment in surface motility was recorded for QS defective mutants in *A. nosocomialis*, a phenotype that could be restored by the addition of OHC12-HSL (Clemmer et al., 2011; Oh and Choi, 2015). The role of QS on motility in *A. baumannii* is less studied, but recently, important differences in motility characteristics have been reported for clinical isolates of *A. baumannii* differing in their antibiotic resistance (López et al., 2017). In *A. baumannii* ATCC^®^ 17978^TM^ (Mayer et al., 2018) and in some but not all the clinical strains studied (López et al., 2017), the addition of the AHL-degrading enzyme Aii20J reduced or even blocked motility completely, supporting a role of AHL-mediated QS on this phenotype in *A. baumannii*, as previously described for *A. nosocomialis*. However, the effect of the mutation of the QS genes on motility in *A. baumannii* and its correlation with biofilm formation has not been studied so far.

*Acinetobacter baumannii* has a strong ability to form biofilms, a capacity that has been linked to this species resistance to desiccation, nutrient starvation, and antimicrobial treatments (Gaddy and Actis, 2009). Potential virulence genes, including those involved in antibiotic resistance, seem to be overexpressed in *A. baumannii* biofilms (Marti et al., 2011; Rumbo-Feal et al., 2017) and a positive correlation was found between biofilm formation and multiple drug resistance in *A. baumannii* clinical isolates (Yang et al., 2019; Zeighami et al., 2019). The infective capacity and the development of chronic infections are related to the capacity to form biofilms (Eze et al., 2018) since genes required for biofilm formation are widespread in *A. baumannii* clinical strains but are partially or completely missing in environmental isolates (Yakkala et al., 2019). Numerous evidence correlates surface-associated motility and biofilm formation in several species and strains of the genus *Acinetobacter* (Stacy et al., 2012; Tucker et al., 2014; Giles et al., 2015; Chen et al., 2017), indirectly indicating that both traits are controlled by QS (Niu et al., 2008; Gaddy and Actis, 2009; Bhargava et al., 2010; Anbazhagan et al., 2012; Stacy et al., 2012; Chen et al., 2017; Rumbo-Feal et al., 2017). A hyper-motile variant of *A. baumannii* ATCC^®^ 17978^TM^ lost its motility and produced less pellicle when two genes of the QS genomic region were mutated (Giles et al., 2015). The reduction of biofilm formation by QQ lactonases has been already demonstrated in clinical isolates of *A. baumannii* (Chow et al., 2014; Zhang et al., 2017) although the effect of these enzymes on biofilm formation was low in comparison with a QS null mutant (Chow et al., 2014). The exogenous addition of the AHL-lactonase Aii20J strongly inhibited biofilm formation in *A. baumannii* ATCC^®^ 17978^TM^ despite the presence of endogenous QQ activity (Mayer et al., 2018), indicating that the endogenous enzymes might be involved in the fine-tuning of the QS system and/or be active during specific periods or conditions. On the contrary, a previous study showed that the AHL-lactonase MomL showed little or no-activity activity on *A. nosocomialis* M2 and several clinical isolates and lost its antibiofilm activity when a QQ-sensitive *A. baumannii* strain was co-cultured with *Pseudomonas aeruginosa* PAO1 (Zhang et al., 2017). It should be noted that most biofilm assays are performed in microtiter plates in which the formation of biofilm by this strictly aerobic species is weak and highly variable. Therefore, to assess the potential use of QQ enzymes for the control of these important virulence traits in *A. baumannii*, it is necessary to assess the effect of the inactivation of *abaI* and the addition of QQ enzymes in conditions that drive to robust biofilm formation and to evaluate the response of different clinical isolates in comparison with *A. baumannii* ATCC^®^ 17978^TM^. Moreover, extracellular DNA (eDNA) is present in *A. baumannii* biofilms and a reduction of biofilm formation by DNase I has been already reported, even in preformed biofilms (Tetz et al., 2009; Sahu et al., 2012) and therefore, the impact of combining QQ strategies with eDNA digestion deserves further exploration.

The key role of QS in the control of virulence factors, including surface-associated motility and biofilm formation in different species/strains of *Acinetobacter* points to the use of QQ enzymes as a possible antimicrobial strategy in these species. Nevertheless, previous studies revealed important differences between species and strains (Vijayakumar et al., 2016; López et al., 2017; Zhang et al., 2017). Therefore, a more detailed study of the role of QS in surface-associated and biofilm formation in *A. baumannii* ATCC^®^ 17978^TM^ and a comparison with clinical strains is required. In order to better understand the influence of QS regulatory mechanisms on these virulence-associated traits in the reference strain *A. baumannii* ATCC^®^ 17978^TM^, in this work, we used a QS defective mutant and QQ strategies. Data presented here reveal that QS regulates both traits in *A. baumannii* ATCC^®^ 17978^TM^ and its behavior differs from *A. nosocomialis* M2. The study of biofilm formation capacity of different multidrug-resistant *A. baumannii* strains and their susceptibility to the QQ enzyme Aii20J and other biofilm-disrupting enzymes revealed a great variability among clinical isolates, indicating that studies targeting the development of novel antimicrobial strategies should be based on multiple strains of different origin and characteristics. Extracellular DNA emerges as a key component of extracellular matrix in *Acinetobacter* biofilms, since the combined action of the QQ enzyme Aii20J and a DNase reduced biofilm formation in all the tested strains.

## Materials and Methods

### Bacterial Strains, Culture Conditions, and Genetic Methods

Bacterial strains and primers used in this study are listed in Table 1. Luria-Bertani (LB) broth and LB agar were used to grow and maintain *Acinetobacter* spp. routinely at 37°C. LB broth was prepared in our laboratory with 1% Bacto-tryptone (Life Technologies Corporation, Thermo Fisher Scientific), 0.5% yeast extract (BD Biosciences) and 1% NaCl (Panreac AppliChem).

**TABLE 1:** Bacterial strains, and primers used in this study.

Strains	Description	Source or reference
*Acinetobacter baumannii*		
ATCC^®^ 17978^TM^		ATCC^*a*^
Δ*abaI* (ATCC^®^ 17978^TM^)	Δ*0109 knock out* (KO) mutant without synthase AHLs gene *abaI* (*A1S_0109*)	This study
Ab1 (ROC013)	*A. baumannii* clinical isolate (respiratory). TM: ST2	López et al., 2017
Ab4 (VAL001)	*A. baumannii* clinical isolate (respiratory). TM: ST169	López et al., 2017
Ab5 (DOM009)	*A. baumannii* clinical isolate (respiratory). TM: ST80	López et al., 2017
Ab7 (HUI001)	*A. baumannii* clinical isolate (respiratory). TM: ST79	López et al., 2017
MAR002	*A. baumannii* biofilm hyper-producing clinical isolate (wound sample) TM: ST271	Álvarez-Fraga et al., 2016
Δ*11085*	MAR002 mutant defective in biofilm formation and cell attachment	Álvarez-Fraga et al., 2016
*Acinetobacter nosocomialis*		
M2		Niu et al., 2008
*abaI:Km* (M2)	*abaI* mutant *(abaI:Km)*, Km^*r*^	Niu et al., 2008
*Escherichia coli*		
BL21(DE3)plysS pET28c(+)-*aii20J*	*E. coli* containing a cloning vector (pET28c(+), Km^*r*^) and *aii20J* gene from *Tenacibaculum* sp. 20J	Mayer et al., 2015
BL21(DE3)plysS pET28c(+)	*E. coli* containing a cloning vector without the *aii20J* gene	Mayer et al., 2015

**Plasmids**	**Reference**	**Use in this work**

pMo130	GenBank: EU862243	Construction of knockout strains

**Primers**	**Sequence (5′–3′)**	**Use in this work**

0109UpFPstI	CCCCTGCAGGGGACTGGTGTCGTTATTACC	Construction of knockout strain Δ*0109*
0109UpREcoRI	GGGGAATTCCCCCTTGGAGTAGAACGTTTATTA	Construction of knockout strain Δ*0109*
0109DownFEcoRI	CCCGAATTCGGGACATAGGCTGTATCGACTT	Construction of knockout strain Δ*0109*
0109DownRBamHI	GGGGGATCCCCCACTGTAGAAATCCCTATACTT	Construction of knockout strain Δ*0109*
0109extF	TGTTCCCGATTATGTATG	Confirmation of Δ*0109*
0109extR	GCAACTTCACAAACTCCA	Confirmation of Δ*0109*
pMo130site2F	ATTCATGACCGTGCTGAC	Checking of pMo130
pMo130site2R	CTTGTCTGTAAGCGGATG	Checking of pMo130

*^*a*^American type culture collection.*

### Construction of Isogenic Deletion Derivatives

Plasmid pMo130, a suicide vector containing the genes *xylE*, *sacB* and a kanamycin resistance marker, was used as described by Hamad et al. (2009). Briefly, 900–1000 bp upstream and downstream regions flanking the genes selected for deletion in *A. baumannii* ATCC^®^ 17978^TM^ were PCR-amplified and cloned into the pMo130 vector using primers listed in Table 1. The resulting plasmid (pMo130-0109) and (pMo130-1750) were transformed into ATCC^®^ 17978^TM^ cells by electroporation (Rumbo-Feal et al., 2013). Recombinant colonies representing the first crossover event were selected by resistance to kanamycin and visual detection of XylE activity following the catechol-based method (Hamad et al., 2009). Bright yellow kanamycin-resistant colonies were then grown overnight in LB supplemented with 15% sucrose and then plated on LB agar without antibiotics. Second crossover event leading to gene deletion was then confirmed by PCR using primers listed in Table 1. The Δ*0109* isogenic deletion and derivative of ATCC^®^ 17978^TM^ was constructed by deleting a region encompassing the *A1S_0109* gene, without affecting the upstream and downstream surrounding genes as described previously (Hamad et al., 2009).

### Motility Assays

Surface-associated motility assays were performed as described by Mayer et al. (2018). Petri dishes were prepared with LB or low-nutrients low-salt LB (0.5% NaCl, 0.2% tryptone, and 0.1% yeast extract; LNLS-LB) media supplemented with 0.25% Difco (Bacto^TM^ Agar) or Eiken agar (Eiken Chemical, Co. Ltd., Japan). Plates with a reduced concentration of NaCl (0.5%), tryptone (0.2%), or yeast extract (0.1%) or media with different concentrations of NaCl (0.1–0.4M) or sucrose (5–20%) were also prepared to determinate the impact of culture media or osmolarity effect on motility, respectively. *N*-Hydroxydodecanoyl-L-homoserine lactone (OHC12-HSL from Sigma, 10 μM), or the AHL-lactonase Aii20J (20 μg/mL) obtained from the marine bacterium *Tenacibaculum* sp. 20J (Mayer et al., 2015) were mixed with the inoculum or with the culture media. One microliter from agitated overnight cultures at 0.3 optical density (OD_600_ nm) was inoculated in the center of the plates. Plates were incubated at 37°C in the dark, and surface-associated motility was inspected after 14 h. Three plates were prepared for each condition, and experiments were repeated at least twice.

### Assessment of Biofilm Formation by *Acinetobacter* spp.

Biofilms were grown in a modification of the *Amsterdam Active Attachment model* (AAA-model) (Exterkate et al., 2010; Muras et al., 2020) assembled with glass coverslips (18 mm × 18 mm). In short, coverslips were vertically submerged in 3 mL of LB or LS-LB (0.5% NaCl, 1% tryptone, and 0.5% yeast extract) inoculated with 24 h-cultures of *A. baumannii* strains at an optical density of 0.05 (OD_600_ nm) in 12-well culture plates. Biofilms were grown at 37°C for 12, 24, or 48 h. The QQ enzyme Aii20J (Mayer et al., 2015) at a final concentration of 20 μg/mL, DNase at 2 U/mL, α-amylase (10 U/mL) were added to the cultures alone or in combination. Wells with only culture medium were incubated in the same conditions as negative growth controls. Three replicates for each strain or treatment were performed. For biofilm biomass quantification, after incubation, the coverslips were removed, deposited in a clean 12-wells culture plate, and allowed to dry in a clean bench. Once dried, wells were filled with Crystal Violet solution (0.04%, Gram-Hucker, Panreac), and after 20 min, the excess of dye was removed, and coverslips washed several times with distilled water. Bound crystal violet was released by adding 33% acetic acid. The absorbance was measured at 600 nm (Muras et al., 2020).

### Transmission Electron Microscopy

Biofilms and planktonic cells of *A. baumannii* ATCC^®^ 17978^TM^, its isogenic mutant Δ*abaI*, and *A. baumannii* ATCC^®^ 17978^TM^ supplemented with 20 μg/mL Aii20J enzyme, were examined by transmission electron microscopy (TEM) to confirm the presence of surface appendages. Coverslips were incubated for 24 h in LS-LB as explained above. Biofilms and planktonic cells obtained by centrifugation (1 mL) were fixed with ice-cold 3.5% paraformaldehyde for 20 min at 4°C. Bacteria were applied to Formvar-coated grids and were air-dried. At least two different formvar coated grids prepared from two different experiments. The cells were then negatively stained with 1% phosphotungstic acid in distilled water for 5 s and were examined with a JEM-1011 transmission electron microscope (JEOL) operating at 80 kV and equipped with an Orius SC1000 charge-coupled device (CCD) camera (Gatan).

### Statistical Methods

Mann–Whitney test (*p* < 0.05) was applied for all statistical analyses using GraphPad Prisma 8.3.0.

## Results

### QS and Surface-Associated Motility in *A. baumannii* ATCC^®^ 17978^TM^

The deletion of gene *A1S_0109*, encoding a putative AHL synthase in *A. baumannii* ATCC^®^ 17978^TM^, showing an amino acid sequence identity of 93.4% with the AbaI synthase of *A. nosocomialis* M2 (Carruthers et al., 2013), caused the disappearance of AHLs from the culture media under the conditions that promoted the production of these signals in the wild-type (Mayer et al., 2018). No differences in growth were observed between the Δ*abaI* mutant and the parental strain in shaken liquid cultures (data not shown).

**FIGURE 1:**
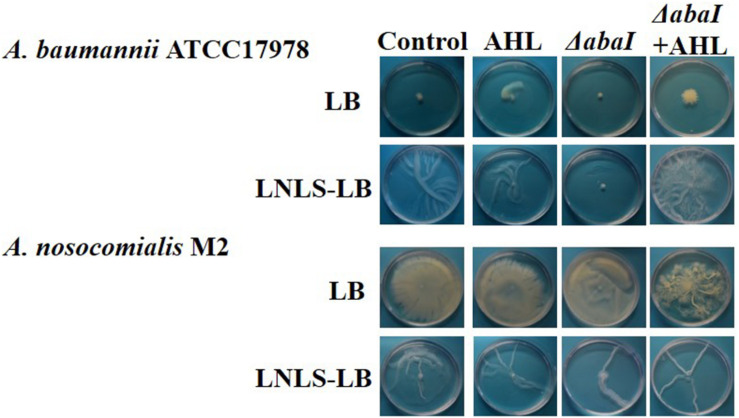
Effect of the mutation of the *abaI* gene on surface-associated motility of *A. baumannii* ATCC^®^ 17978^TM^, and *A. nosocomialis* M2. The capacity of OHC12-HSL (10 μM) to restore the phenotype in the mutants was tested. Plates were prepared in LB or low-nutrient low-salt LB (LNLS-LB) with 0.25% Eiken agar and were incubated at 37°C for 14 h. Images are representative of three independent experiments carried out with three replicates per condition.

LB medium completely hindered surface-associated migration in *A. baumannii* ATCC^®^ 17978^TM^ (Figure 1), while a “tentacle-like,” branched motility pattern was observed in LNLS-LB for this species at 37°C. This phenotype was not affected by the type of agar used (Supplementary Figure 1). On the contrary, *A. nosocomialis* M2 maintained the motility in both LB and LNLS-LB (Figure 1) but only on plates prepared with Eiken agar (Supplementary Figure 1). Deletion of the *abaI* gene completely abolished motility under permissive conditions in *A. baumannii* ATCC^®^ 17978^TM^ while almost no effect could be observed in the *A. nosocomialis* M2 *abaI:Km* mutant at 37°C (Figure 1). The exogenous addition of OHC12-HSL, the major AHL signal found in the supernatants of *A. baumannii* ATCC^®^ 17978^TM^ and *A. nosocomialis* M2 cultures (Niu et al., 2008; Mayer et al., 2018) was able to restore this phenotype in the Δ*abaI* mutant of ATCC^®^ 17978^TM^ under permissive conditions, confirming that AHL-mediated QS is crucial for surface-associated motility in this species, while no significant effect was observed when added to the wild type (Figure 1).

**FIGURE 2:**
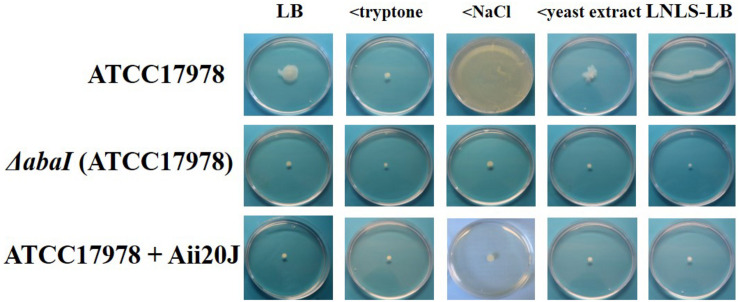
Effect of the components of LB culture medium on surface-associated motility of *A. baumannii* ATCC^®^ 17978^TM^ and its isogenic mutant Δ*abaI*, on different media plates with 0.25% Eiken agar. The effect of the QQ enzyme Aii20J (20 μg/mL) on *A. baumannii* ATCC^®^ 17978^TM^ was also evaluated. Plates were incubated at 37°C for 14 h and images are representative of three independent experiments carried out carried out with three replicates per condition (< tryptone: LB with reduced tryptone concentration, < NaCl: LB with reduced NaCl concentration, < yeast extract: LB with reduced yeast extract concentration, and LNLS-LB: low-nutrient low-salt LB).

A systematic study of plate motility was performed by reducing the concentrations of each LB medium component to that of LNLS-LB to define better the LB-medium component responsible for abrogating surface-associated motility in *A. baumannii* ATCC17978 and its effect on the role of the QS system. The experiments were performed in Eiken agar, that was shown to be more motile-permissive for *A. nosocomialis* M2 strain (Supplementary Figure 1). Results showed that a reduction in salt concentration from 1 to 0.5% while keeping other LB constituent concentrations, promotes a hyper-motile phenotype in ATCC^®^ 17978^TM^ (Figure 2) similar to that observed in M2 in LB medium (Figure 1 and Supplementary Figure 2). Lowering tryptone or yeast extract concentrations did not change the non-motile phenotype observed in LB (Figure 2), indicating that NaCl concentrations higher than 0.5% are responsible for the inhibition of motility observed in LB medium for ATCC^®^ 17978^TM^. Additional experiments carried out by substituting NaCl by sucrose indicated that high osmolarity conditions, and not simply ionic strength, are responsible for the abrogation of motility in this strain (Supplementary Figure 3). In the case of *A. nosocomialis* M2, the reduction of tryptone, the main source of C and N in LB medium, was responsible for the change in the motility pattern observed in LNLS-LB (Figure 1), with no effect of NaCl concentration (Supplementary Figure 2). The loss of surface-associated motility capacity in the Δ*abaI* mutant of ATCC^®^ 17978^TM^ was confirmed in all the tested media (Figure 2). The exogenous addition of the QQ enzyme Aii20J, mixed with the inoculum, had a similar effect that the mutation of the synthase, completely blocking motility (Figure 2) in LNLS-LB plates. On the contrary and in accordance with the higher production of AHLs that has been reported in low-salt LB liquid cultures in comparison with LNLS-LB (Mayer et al., 2018), in the case of the low-salt LB-plates, mixing the enzyme with the inoculum was not enough to block the motility, and it was necessary to mix the enzyme with all the culture media in order to fully abrogate this phenotype. At 37°C the *abaI*:*Km* mutant of *A. nosocomialis* M2 presented the same spreading phenotype as the parental strain in all culture media (Supplementary Figure 2).

### Biofilm Formation Is Under the Control of QS in *A. baumannii* ATCC^®^ 17978^TM^

**FIGURE 3:**
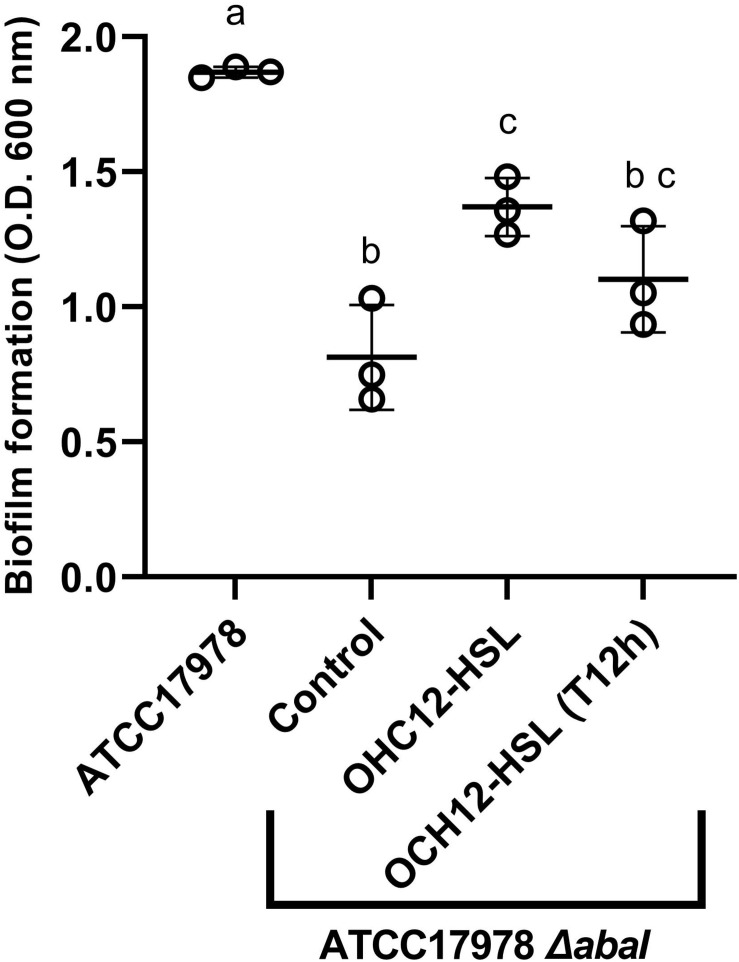
Biofilm formation by *A. baumannii* ATCC^®^ 17978^TM^ and its Δ*abaI* mutant as measured with crystal violet staining. The capacity of OHC12-HSL (10 μM) to restore the phenotype in the mutant was also tested. The addition of OHC12-HSL was performed at the beginning of the experiment and after 12 h of incubation (T12h). Biofilms (*n* = 3) were inoculated in LS-LB, incubated at 37°C for 24 h and stained with crystal violet staining. Different letters indicate statistically significant differences (Mann–Whitney test, *p* < 0.05).

A preliminary experiment was performed in order to assess if low salinity promoted biofilm formation in *A. baumannii* ATCC^®^ 17978^TM^, as observed for surface-associated motility. Results clearly show a significantly higher biofilm formation (Mann–Whitney test, *p* < 0.05) in ATCC^®^ 17978^TM^ in the LS-LB medium in the first 12 h, while no differences were observed thereafter with the crystal violet assay quantification (Supplementary Figure 4). The mutation of *abaI* gene caused a strong reduction in biofilm formation in *A. baumannii* ATCC^®^ 17978^TM^ in both culture media (Supplementary Figure 4). In an experiment performed in LS-LB, the addition of the QS signal OHC12-HSL to the mutant partially restored the phenotype, causing an increase of the biofilm biomass, that was statistically significant (Mann–Whitney test, *p* < 0.05, Figure 3). The addition of the AHL to a preformed-biofilm (T12h) of the mutant also produced an increase in biofilm biomass, although this difference was not statistically significant (Mann–Whitney test, *p* > 0.05).

### Effect of the QQ Enzyme Aii20J and Other Biofilm Inhibitory Enzymes on Biofilm Formation by *A. baumannii* ATCC^®^ 17978^TM^

**FIGURE 4:**
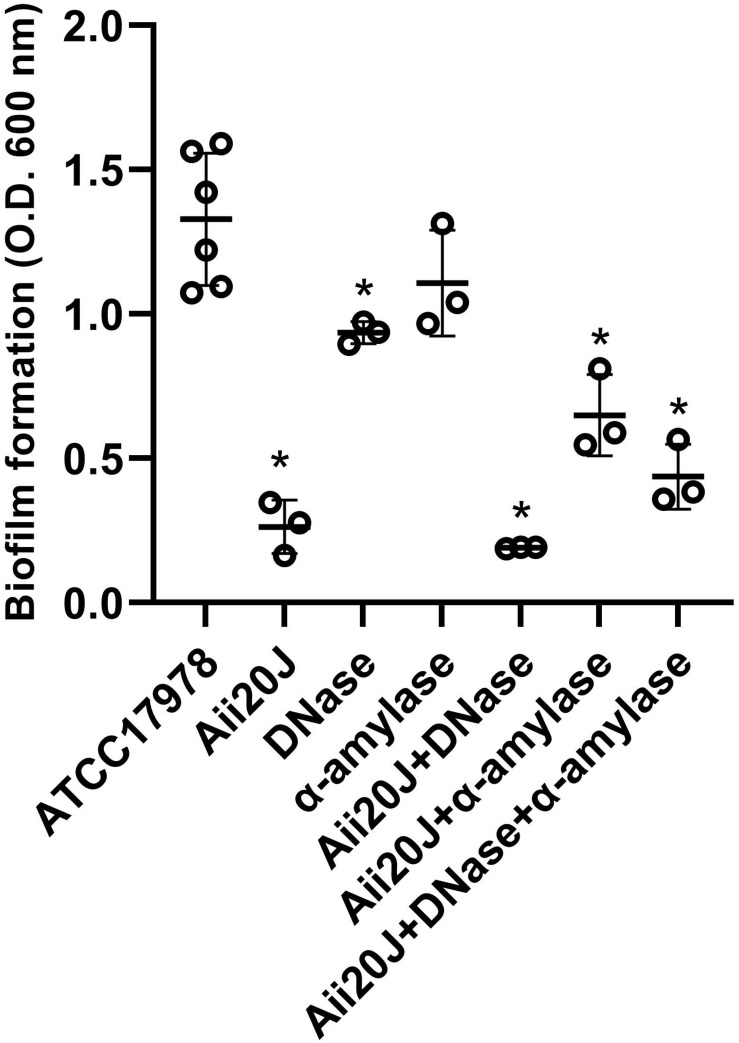
Effect of different enzymes on biofilm formation by *A. baumannii* ATCC^®^ 17978^TM^. The QQ enzyme Aii20J (20 μg/mL), DNase (2 U/mL), and α-amylase (10 U/mL) were added alone or in combination. Biofilms were produced in LS-LB in glass coverslips, incubated for 24 h at 37°C and stained with the crystal violet assay. The cultures were inoculated with cells from an agitated culture. Values are averages ± SD (*n* = 3). Asterisks indicate statistically significant differences in comparison with the untreated control (Mann–Whitney test, *p* < 0.05).

The purified QQ enzyme Aii20J reduced biofilm formation in *A. baumannii* ATCC^®^ 17978^TM^ by 80% after 24 h when grown on glass coverslips at 37°C (Figure 4). The enzyme also affected biofilm formation in *A. nosocomialis* M2 in the same system, although the reduction was lower (38.54%). The clinical strain MAR002 and an isogenic mutant impaired in biofilm formation and eukaryotic-cell attachment capacity were equally affected by the enzyme, suffering a reduction of biofilm formation of around 45% (Supplementary Figure 5). Biofilm formation in MAR002 and its mutant was similar to that observed for ATCC^®^ 17978^TM^, even though MAR002 has been described as a biofilm hyper-forming strain (Álvarez-Fraga et al., 2016).

The effect of the purified QQ enzyme Aii20J was compared with the effect of two enzymes with potential biofilm inhibitory effect: a DNase and an α-amylase. The α-amylase did not affect biofilm formation by ATCC^®^ 17978^TM^ (Mann–Whitney test, *p* = 0.16, Figure 4). On the contrary, DNase significantly reduced biofilm formation by 20% (Mann–Whitney test, *p* < 0.05, Figure 4). In ATCC^®^ 17978^TM^ the simultaneous addition of the QQ enzyme Aii20J and DNase did not improve significantly the results obtained with the QQ enzyme (Mann–Whitney test, *p* = 0.35, Figure 4).

**FIGURE 5:**
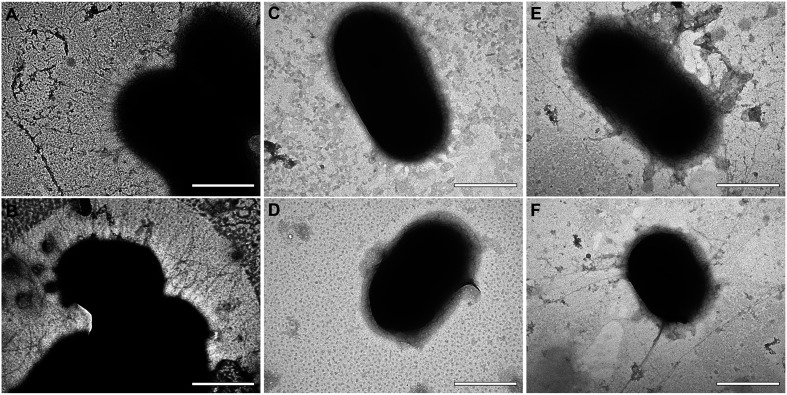
TEM microphotographs of *A. baumannii* ATCC^®^ 17978^TM^ **(A,B)**, its isogenic mutant Δ*abaI* **(C,D)** and *A. baumannii* ATCC^®^ 17978^TM^ treated with the QQ enzyme Aii20J (20 μg/mL) **(E,F)**. Original magnification: 80.000×. Scale bars: 500 nm.

In order to observe the effect of Aii20J on cell appendages, biofilm and planktonic cells were observed with TEM. Important differences could be observed among wild type, the Δ*abaI* mutant and the cells treated with the pure Aii20J enzyme (Figure 5). *A. baumannii* ATCC^®^ 17978^TM^ cells showed many short appendages (Figures 5A,B). On the contrary, cells of the Δ*abaI* mutant and Aii20J-treated cells were almost completely devoid of these short appendages (Figures 5C–F). Cells surrounded by long, thin individual filaments were observed in the cultures in the presence of the Aii20J enzyme (Figures 5E,F). The same morphology was observed in attached and unattached cells (data not shown).

### Effect of the QQ Enzyme Aii20J and DNase on Biofilm Formation by Multidrug-Resistant Clinical Isolates of *A. baumannii*

**FIGURE 6:**
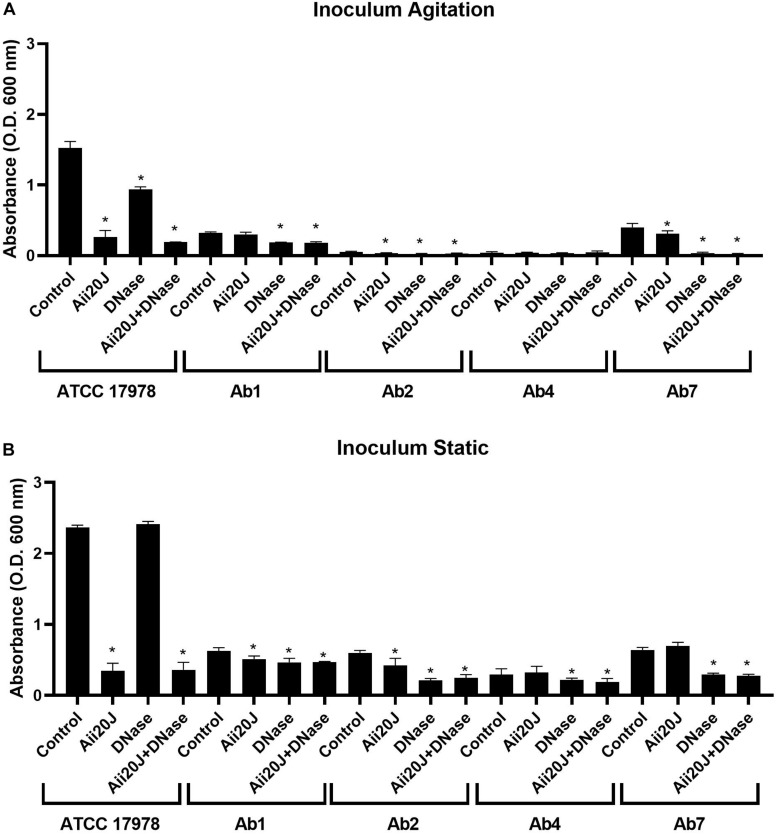
Effect of the QQ enzyme Aii20J (20 μg/mL), DNase (2 U/mL) or both on biofilm formation by *A. baumannii* ATCC^®^ 17978^TM^ and different multidrug-resistant clinical strains presenting different motility patterns (López et al., 2017). Biofilms were cultivated on 18 mm × 18 mm glass coverslips in LS-LB during 24 h at 37°C and stained with crystal violet (*n* = 3). The cultures were inoculated with cells from an agitated **(A)** or a static **(B)** culture. Asterisks indicate statistically significant differences in comparison with the untreated control (Mann–Whitney test, *p* < 0.05).

Several clinical isolates that had been previously characterized regarding antibiotic-resistance profile, AHL production and surface-associated motility (López et al., 2017; Mayer et al., 2018) were selected and tested regarding its capacity to form biofilm on glass coverslips and their sensitivity to the enzymes Aii20J and DNase. Large differences in biofilm formation were found in all strains depending on the incubation conditions of the inoculum, with much higher biofilm formation being observed when the inoculum was maintained under static conditions (Figure 6B). Moreover, important differences among the tested strains were observed. *A. baumannii* ATCC^®^ 17978^TM^ formed much more biofilm than any of the clinical MDR strains, independently of the culture conditions of the inoculum (Figures 6A,B). Among these, the biofilm formation capacity was not related to the antibiotic resistance or surface-associated motility profiles. Strains with high motility profile (Ab1, Ab5, Ab7) showed low or intermediate biofilm formation capacity *in vitro*. The effect of the enzymes was also variable among the strains (Figures 6A,B). However, no significant effect on bacterial growth, measured as optical density in the supernatant of the cultures was observed, except for ATCC17978 in which OD in the supernatants in the presence of the enzymes was significantly higher than in the control culture (data not shown). Strains ATCC^®^ 17978^TM^ and Ab7, showing a very similar surface-associated motility pattern and response to the QQ enzyme (López et al., 2017) showed a very different response regarding biofilm formation. Best inhibitory results were obtained with the combination of the QQ enzyme and DNase in all cases (Figures 6A,B).

## Discussion

The study of the factors controlling the expression of virulence factors is crucial for the development of novel antimicrobial strategies in antibiotic-resistant pathogens like *A. baumannii*. Multiple transcriptional regulatory systems are involved in the persistence and pathogenesis of this species (Wood et al., 2018a; De Silva and Kumar, 2019). Among these regulatory circuits, QS has emerged as an interesting target for the control of virulence factors (Zhang et al., 2017; Mayer et al., 2018). Most studies regarding the role of the AHL-mediated QS system in the expression of virulence-related traits have been carried out with *A. nosocomialis* strain M2 (formerly *A. baumannii* M2) (Niu et al., 2008; Bhargava et al., 2010; Clemmer et al., 2011; Stacy et al., 2012, 2013; Saroj and Rather, 2013; Oh and Choi, 2015). The role of the QS system in this intricate signaling system seems to be crucial, since the mutation of the AbaI synthase significantly reduces virulence in *in vivo* models (Peleg et al., 2009; Fernández-García et al., 2018). Nevertheless, in the view that some important features, such as motility, behave differently between *A. nosocomiali*s M2 and *A. baumannii* ATCC^®^ 17978^TM^ (Clemmer et al., 2011), it is important to fully characterize the role of QS system in the latter and to study the variations among clinical strains of *A. baumannii*. As previously reported for other species of the genus (Niu et al., 2008; Bitrian et al., 2012; How et al., 2015), the mutation of the *abaI* gene (A1S_0109) fully abolished AHL production in *A. baumannii* ATCC^®^ 17978^TM^, indicating that this gene probably codifies the only synthase responsible for AHL-mediated QS in this species.

Both, motility (Figures 1, 2) and biofilm formation (Figure 3) were impaired in the Δ*abaI* mutant in *A. baumannii* ATCC^®^ 17978^TM^, corroborating previous reports on the control of these two important traits related to virulence by the AHL-mediated QS system in other *Acinetobacter* spp. (Niu et al., 2008; Kang and Park, 2010; Anbazhagan et al., 2012; Bhargava et al., 2012; Kim and Park, 2013; Chow et al., 2014; Oh and Choi, 2015; Mayer et al., 2018). The exogenous addition of OHC12-HSL, the main AHL found in *Acinetobacter* spp., restored the motility of the Δ*abaI* mutant under motile-permissive, low-salt conditions (Figure 1) and partially restored biofilm forming capacity (Figure 3). Consistent with these outcomes, in ATCC^®^ 17978^TM^ the wide-spectrum AHL-lactonase Aii20J blocked motility even in highly inductive conditions (Figure 2). Moreover, it drastically reduced biofilm formation (Figures 4, 6 and Supplementary Figure 5), confirming a key role of QS in the regulation of these two traits in this strain and further supporting the potential of QQ strategies for virulence control in *Acinetobacter* (Stacy et al., 2013; Chow et al., 2014; Mayer et al., 2015, 2018, 2020; López et al., 2017; Zhang et al., 2017). Nevertheless, a wide variability in surface-associated motility and response to QQ strategies has been previously described in *A. baumannii* clinical isolates presenting different antibiotic resistance profiles (López et al., 2017). Also, the well-studied strain *A. nosocomialis* M2 presented very different behavior in response to culture conditions and QQ strategies in comparison with ATCC^®^ 17978^TM^, maintaining a strong motility phenotype in conditions in which *A. baumannii* ATCC^®^ 17978^TM^ motility was completely blocked (Supplementary Figure 2). Unlike what was observed for the QS-impaired mutant of ATCC^®^ 17978^TM^, the *A. nosocomialis* M2 *abaI:Km* mutant maintained the motility pattern at 37°C in contrast with the impairment of motility reported at 30°C (Clemmer et al., 2011) and confirmed in our laboratory (data not shown), indicating a strong effect of temperature in this type of *in vitro* assays. Our results demonstrate that surface-associated motility in M2 is not so strongly dependent on QS regulatory pathways, at least in motile-permissive conditions, revealing important differences in the role of QS on this phenotype among species and isolates of the genus *Acinetobacter*.

High salinity negatively affected both surface-associated motility and initial biofilm formation in ATCC^®^ 17978^TM^ (Figure 2 and Supplementary Figure 4). Salinity lost its influence during biofilm maturation under the conditions tested (Figure 2), indicating that the factors that favor motility at low salinities are important for the initial steps of biofilm formation, and linking the mechanisms responsible for surface-associated motility with the surface-attachment phase of biofilm formation. Indeed, AHL production is higher in low-salinity liquid cultures (Mayer et al., 2018), which also correlates QS with these two physiological processes. In low-salt solid cultures it was necessary to increase the concentration of the QQ enzyme Aii20J in order to reduce motility, indicating a higher production of QS signals also in semi-solid LS-LB. High osmolarity was shown to repress motility but to activate biofilm formation in *Vibrio cholerae* (Shikuma and Yildiz, 2009), suggesting that medium osmolarity is an important environmental signal regulating lifestyle in biofilm-forming pathogens.

The electron microscope analysis of cells obtained during early biofilm formation shows a clear decrease in surface short pili in the Δ*abaI* mutant (Figure 5), which could be the result of lower expression of the *csu* operon. The chaperone/usher pili-associated *csu* operon, responsible for the assembly and extension of type I pilus (Tomaras et al., 2003, 2008), important for biofilm formation in *A. nosocomialis* M2 and *A. baumannii* ATCC^®^ 19606^TM^ (Tomaras et al., 2008; Gaddy and Actis, 2009; Clemmer et al., 2011; Luo et al., 2015), has been previously described to be overexpressed in biofilms in comparison with planktonic cultures of *A. baumannii* ATCC^®^ 17978^TM^ (Rumbo-Feal et al., 2013). The *csu* operon has also been related to surface-associated motility in *A. baumannii* ATCC^®^ 17978^TM^ since the conditions that promote motility and AHL signal production also generate an increase in the expression of *csuD* (Mayer et al., 2018). The mutation of A1S_2811, a CheA/Y-like hybrid, a two-component regulator in *A. baumannii* ATCC^®^ 17978^TM^, resulted in a decrease in surface motility and biofilm formation at the gas-liquid interface and the transcription of *abaI* and the *csu* operon. The reduction of biofilm and motility in the mutant was accompanied by a decrease in pilus-like structures and exopolymeric substances on the cell surface (Chen et al., 2017). Moreover, Luo et al. (2015) showed that addition of AHL signals to *A. baumannii* ATCC19606^TM^ cultures induces the expression of bacterial pili structural genes, including *csuD*, and an increase of pili-like structures around the bacteria. Our results further support a role of the *csu* operon in surface-associated motility and biofilm formation, both being under the control of the QS signals in ATCC^®^ 17978^TM^. Consistent with a control of *csu* expression by the QS system, short pili were also absent in cells treated with the QQ enzyme Aii20J; instead, long filaments were found around the cells treated with the enzyme, that were absent in the wild type and in the Δ*abaI* mutant (Figure 5), indicating that the QQ enzyme could not block the production of a different type appendages. These filaments could be attributed, among others, to type IV pili (TFP), involved in several bacterial processes such as natural transformation or adherence to biotic and abiotic surfaces and twitching motility (Clemmer et al., 2011; Harding et al., 2013; Eijkelkamp et al., 2014). However, the involvement of TFP in surface-associated motility and its regulation by the QS system is controversial (reviewed by Harding et al., 2018). A novel photo-regulated type I chaperone/usher pilus assembly system, encoded by the *prpABCD* operon, involved in surface-associated motility, biofilm formation and virulence to *Galleria mellonella* (Wood et al., 2018b) could be also responsible for the observed phenotype in the Aii20J-treated cells. Additionally, the synthesis of 1,3-diaminopropane (DAP), lipid oligosaccharides, other external envelope components or extracellular DNA, important for motility and biofilm formation in this species (McQueary et al., 2012; Harding et al., 2018; Geisinger et al., 2019) may also be under the control of the QS system, contributing to the observed effect of the mutation of the *aba*I gene and Aii20J lactonase on both, surface-associated motility and biofilm formation.

The status of the inoculum strongly influenced the formation of biofilm in the *Amsterdam Active* Attachment model (Figure 6). More biofilm was formed when the inoculum was maintained in static conditions, a phenomenon already reported for pellicle formation in the liquid-air interface and motility in *A. baumannii* ATCC^®^ 17978^TM^ (Chen et al., 2017). This fact also links biofilm formation with the QS system, since AHLs are only produced in significant amounts in static cultures (Mayer et al., 2018). Therefore, the cells maintained in such conditions should display a set of physiological activities that favor or accelerate the initiation of biofilm formation when transferred to fresh medium.

As previously reported for surface-associated motility (López et al., 2017), strong variations in biofilm formation were found among the different strains tested, and no clear correlation was found between strain origin, antibiotic resistance profile and *in vitro* biofilm formation ability (Figure 6). A large number of works have analyzed the correlation between biofilm formation and antibiotic resistance in *A. baumannii* (reviewed by Eze et al., 2018; Mayer et al., 2020). Biofilm formation has been found to be correlated with MDR among clinical isolates (Babapour et al., 2016; Yang et al., 2019; Zeighami et al., 2019). Higher incidence of antibiotic resistance has also been associated with the presence of *abaI* and *csuE* genes in clinical *A. baumannii* strains (Liu et al., 2016). On the contrary, another study could not find any relationship between isolation site, multidrug resistance, pulsed-field type and biofilm production among *A. baumannii* clinical and environmental strains isolated from a hospital (de Campos et al., 2016). Finally, it was reported that isolates with a higher level of resistance tended to form weaker biofilms (Qi et al., 2016) driving to the hypothesis that biofilm-forming bacterial strains do not depend on antibiotic resistance and colonization characteristics as do non-biofilm formers for their survival in hospital settings (Hall and Mah, 2017). In our case, the low-antibiotic resistant strain ATCC^®^ 17978^TM^, isolated from a case of infant meningitis, shows a much higher biofilm-forming capacity than the MDR strains (Figure 6).

The QQ lactonase Aii20J strongly reduced biofilm formation in *A. baumannii* ATCC^®^ 17978^TM^. This fact indicates that the many existing QQ enzymes present in this strain may be active only under particular conditions or during specific periods, probably related to the self-control of AHL production during stationary phase (Mayer et al., 2018). The lactonase was also capable of reducing biofilm formation in *A. nosocomialis* M2 and in MAR002, that was previously reported as a biofilm hyper-forming strain, even though in our cultivation system the amount of biofilm formed was similar to that observed in *A. baumannii* ATCC^®^ 17978^TM^ (Supplementary Figure 5). This difference is probably the result of the different cultivation methodology and culture medium used, since in tubes, these strains form biofilm only in the interphase liquid:air (Tomaras et al., 2003). The lactonase Aii20J also reduced biofilm formation significantly in two of the four MDR clinical strains tested (Figure 6). Sensitivity to the enzymes in the clinical MDR strains was not dependent of MDR profile or biofilm-forming capacity. However, it could be somehow related to the amount of AHLs produced since strains Ab5 and Ab7 produced a negligible amount of AHLs in LB medium at 37°C (Mayer et al., 2018) and demonstrated no-sensitivity to the action of the QQ enzyme (Figure 6). A recombinant lactonase successfully disrupted the biofilm formation of clinical isolates of *A. baumannii* (Chow et al., 2014). However, MomL was ineffective in treating mixed species and wound-associated biofilm models containing *A. baumannii* (Zhang et al., 2017). The differences observed in biofilm formation when the inoculum was maintained in static or shaken conditions indicate that the published results regarding biofilm formation may be strongly influenced by cultivation conditions and should be interpreted carefully. Temperatures lower than 30°C seem to favor biofilm formation (Eze and El Zowalaty, 2019), and QS regulated genes that are relevant for biofilm formation are upregulated at lower temperatures (De Silva and Kumar, 2019). Current biofilm experiments were carried out at 37°C, and therefore the effect of the QQ lactonase Aii20J may be higher at lower temperatures. Also, the weak biofilm formation by this species in the microtiter-plate method generally used to cultivate the biofilms, and the low sensitivity of the crystal-violet-staining method used for the assessment of biofilm biomass in most studies should be taken into account when evaluating anti-biofilm activity (Muras et al., 2018).

Despite the variability in response to the QQ lactonase Aii20J and DNase, the simultaneous addition of both enzymes reduced biofilm formation in the five strains tested, and therefore constitutes a promising strategy for controlling biofilm formation in this species. The presence of eDNA in *A. baumannii* biofilms has been reported before in a clinical isolate and a reduction of biofilm formation by DNase I has been already reported, even in preformed biofilms (Tetz et al., 2009; Sahu et al., 2012). Since the sequence of eDNA is similar to genomic DNA, the production of eDNA may also constitute a way of transferring antibiotic resistance in this species (Li et al., 2008). eDNA has been reported in numerous Gram-positive and Gram-negative bacterial pathogens, and the efficacy of the use of DNase has been demonstrated in many of them, increasing the efficacy of antibiotics (Tetz et al., 2009; Okshevsky et al., 2015). In our case, the efficacy of DNase to reduce biofilm formation in *A. baumannii* ATCC^®^ 17978^TM^ and different clinical isolates was highly strain-dependent (Figure 6). In the view of the obtained results, the pathway of eDNA secretion in *A. baumannii* deserves further investigation since it constitutes a promising target for developing combined antibiofilm strategies.

Based on the study of the Δ*abaI* mutant and the action of the QQ enzyme Aii20J, results confirm a central role of the QS in the control of biofilm formation and surface-associated motility in *A. baumannii* ATCC^®^ 17978^TM^. This role seems to be strain-specific and strongly dependent on the cultivation conditions. Results demonstrate that QQ strategies, in combination with other enzymatic treatments such as DNase, could represent an alternative approach for the prevention of *A. baumannii* colonization and survival of surfaces and the prevention and treatment of infections caused by this pathogen. The wide variability observed in surface-associated motility, biofilm forming capacity and sensitivity to the QQ enzyme among the tested *A. baumannii* isolates indicate that the development of any novel antimicrobial strategy targeting virulence traits should be carefully evaluated on a large number of strains.

## Data Availability Statement

All datasets generated for this study are included in the article/Supplementary Material.

## Author Contributions

CM, AM, and AP conducted the experiments and contributed to the writing of the manuscript. SR-F constructed the mutant. JR-V performed the TEM analysis and contributed to the writing of the manuscript. MR and MP contributed to the writing of the manuscript. AO designed the experiments and wrote the manuscript. All authors contributed to the article and approved the submitted version.

## Conflict of Interest

The enzyme Aii20J, used in some experiments described in this work, is protected by the following patent: AO, MR, and CM (2016). Peptide with quorum-sensing inhibitory activity, polynucleotide that encodes said peptide, and the uses thereof. PCT/ES2014/070569. The remaining authors declare that the research was conducted in the absence of any commercial or financial relationships that could be construed as a potential conflict of interest.

## Supplementary Material

Supplementary Figure 1 Effect of culture media composition and type of agar on surface-associated motility of *A. baumannii* ATCC17978, *A. nosocomialis* M2 and their respective mutants of the AbaI AHL-synthase. LB and Low Salt-Low Nutrient LB (LSLN-LB 0.5% NaCl, 0.2% tryptone, and 0.1% yeast extract) were tested. Eiken or Difco agar were added at 0.25%. Plates were incubated at 37°C for 14 h, and they are representative of three independent experiments carried out with three replicates per condition.

Supplementary Figure 2 Effect of the components of LB culture medium on the motility of *A. nosocomialis* M2 and its *abaI:Km* mutant. 0.25% Eiken agar plates were incubated at 37°C for 14 h. Images are representative of three independent experiments carried out with three replicates per condition.

Supplementary Figure 3 Effect of osmolarity on surface-associated motility in *A. baumannii* ATCC^®^ 17978^TM^ **(A)** and *A. nosocomialis* M2 **(B)**. Cells were inoculated on LB plates with different NaCl (0.1–0.4M) or sucrose (5–20%) concentrations. Plates were incubated at 37°C for 14 h. Images are representative of three independent experiments carried out carried out with three replicates per condition. NaCl concentration in standard LB medium is 0.17M.

Supplementary Figure 4 Biofilm formation by *A. baumannii* ATCC^®^ 17978^TM^ and its isogenic mutant Δ*abaI* in LB (gray bars) and low-salt LB (LS-LB, NaCl 0.5%, white bars) culture media. Biofilms were formed on 18 mm × 18 mm glass coverslips using a modification of the *Active Attachment* model (Exterkate et al., 2010), incubated for 24 h at 37°C and quantified using the crystal violet assay. Values are shown as average ± SD (*n* = 3). The asterisk shows statistically significant differences (Mann–Whitney test, *p* < 0.05) between LB and LS-LB.

Supplementary Figure 5 Effect of the QQ lactonase Aii20J (20 μg/mL) on biofilm formation by *A. baumannii* ATCC^®^ 17978^TM^, the *A. baumannii* clinical strain MAR002, which was reported to be a biofilm hyper-former, its isogenic mutant Δ11085, which was reported to be defective in biofilm formation and eukaryotic cell attachment in comparison with the parental strain (Álvarez-Fraga et al., 2016) and *A. nosocomialis* M2. **(A)** Biofilm quantification using the Crystal Violet staining method. Values are averages ± SD (*n* = 3). The asterisks show statistically significant differences (Mann–Whitney test, *p* < 0.05) between treated and untreated biofilms for the same strain. **(B)** Pictures showing the aspect of the untreated (left) and treated (right) biofilms stained with crystal violet. Biofilms were produced on 18 mm × 18 mm glass coverslips using a modification of the *Amsterdam Active Attachment* model (Exterkate et al., 2010) in LS-LB, incubated for 24 h at 37°C.
