# Supplementary material for: Quorum Sensing as a Target for Controlling Surface Associated Motility and Biofilm Formation in Acinetobacter baumannii ATCC® 17978TM
Source: Front Microbiol. 2020 Sep 30;11:565548. doi: 10.3389/fmicb.2020.565548 (PMC7554515; doi:10.3389/fmicb.2020.565548)
Supplement: Supplementary Figure 1 — Effect of culture media composition and type of agar on surface-associated motility of A. baumannii ATCC17978, A. nosocomialis M2 and their respective mutants of the AbaI AHL-synthase. LB and Low Salt-Low Nutrient LB (LSLN-LB 0.5% NaCl, 0.2% tryptone, and 0.1% yeast extract) were tested. Eiken or Difco agar were added at 0.25%. Plates were incubated at 37°C for 14 h, and they are representative of three independent experiments carried out with three replicates per condition. [file Data_Sheet_1.PDF]

## Quorum sensing as a target for controlling surface associated motility and biofilm formation in *Acinetobacter baumannii* ATCC®17978™

**Celia Mayer, Andrea Muras, Ana Parga, Manuel Romero, Soraya Rumbo-Feal,**

**Margarita Poza, José Ramos-Vivas, Ana Otero**

# *Supplementary Material*

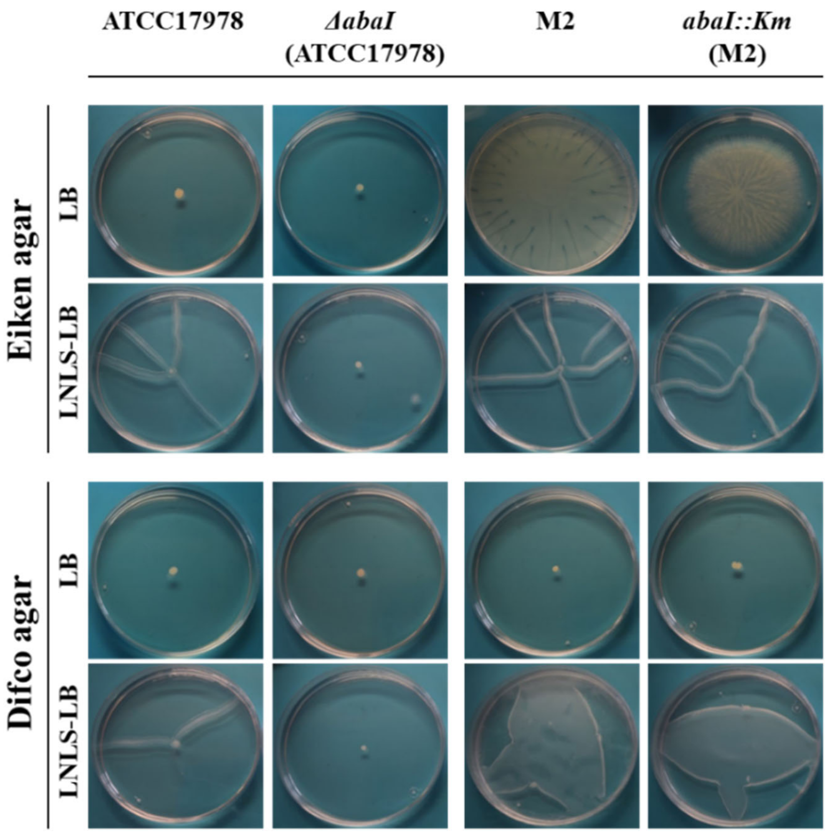


**Supplementary Figure 1.** Effect of culture media composition and type of agar on surface-associated motility *of A. baumannii* ATCC17978, *A. nosocomialis* M2 and their respective mutants of the AbaI AHL-synthase. LB and Low Salt-Low Nutrient LB (LSLN-LB 0.5% NaCl, 0.2% tryptone and 0.1% yeast extract) were tested. Eiken or Difco agar were added at 0.25%. Plates were incubated at 37ºC for 14 h and are representative of 3 independent experiments carried out carried out with 3 replicates per condition.

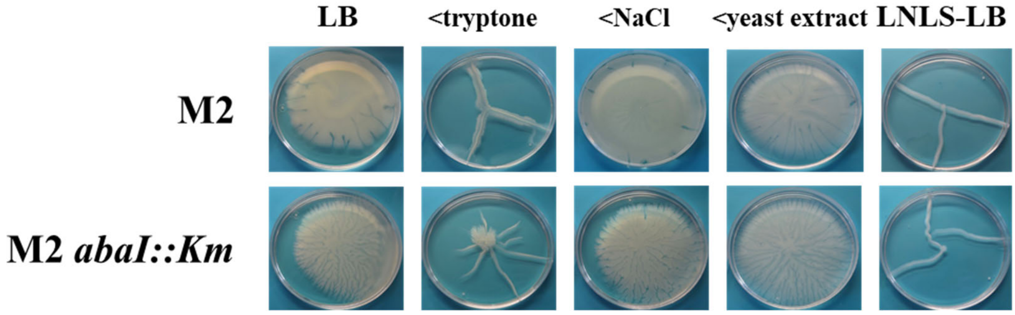


**Supplementary Figure 2**. Effect of the components of LB culture medium on motility of *A. nosocomialis* M2 and its *abaI::Km* mutant. LB was prepared with 0.25% Eiken agar. Plates were incubated at 37ºC for 14 h. Images are representative of 3 independent experiments carried out carried out with 3 replicates per condition.

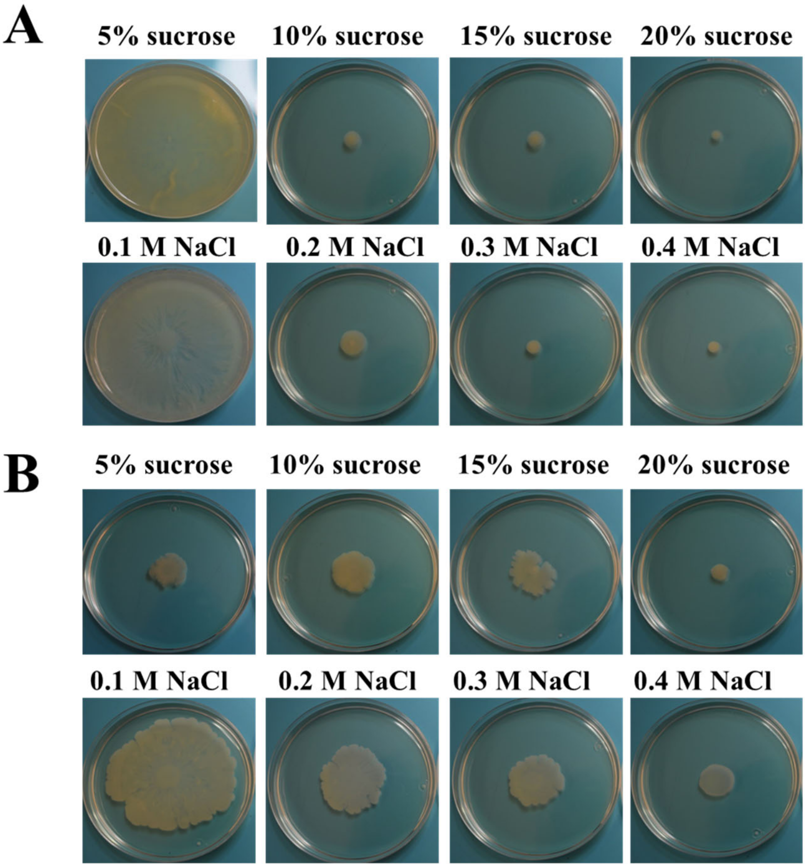


**Supplementary Figure 3.** Effect of osmolarity on surface-associated motility in *A. baumannii* ATCC® 17978™ (A) and *A. nosocomialis* M2 (B). Cells were inoculated on LB plates with different NaCl (0.1-0.4 M) or sucrose (5-20%) concentrations. Plates were incubated at 37ºC for 14 h. Images are representative of 3 independent experiments carried out with 3 replicates per condition. NaCl concentration in standard LB medium is 0.17 M.

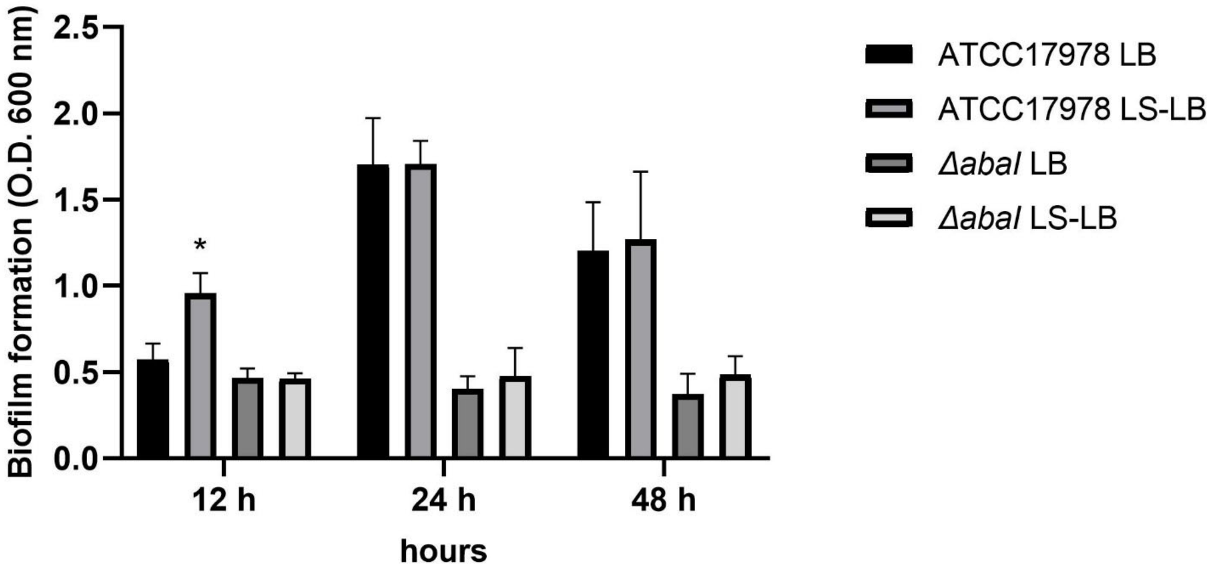


**Supplementary Figure 4.** Biofilm formation by *A. baumannii* ATCC® 17978™ and its isogenic mutant *ΔabaI* in LB (grey bars) and low-salt LB (LS-LB, NaCl 0.5%, white bars) culture media. Biofilms were formed on 18x18 mm glass coverslips using a modification of the *Active Attachment* model (Exterkate et al. 2010), incubated for 24 h at 37ºC and quantified using the crystal violet assay. Values are shown as average ± sd (n=3). The asterisk shows statistically significant differences (Mann-Whitney test, P<0.05) between LB and LS-LB.

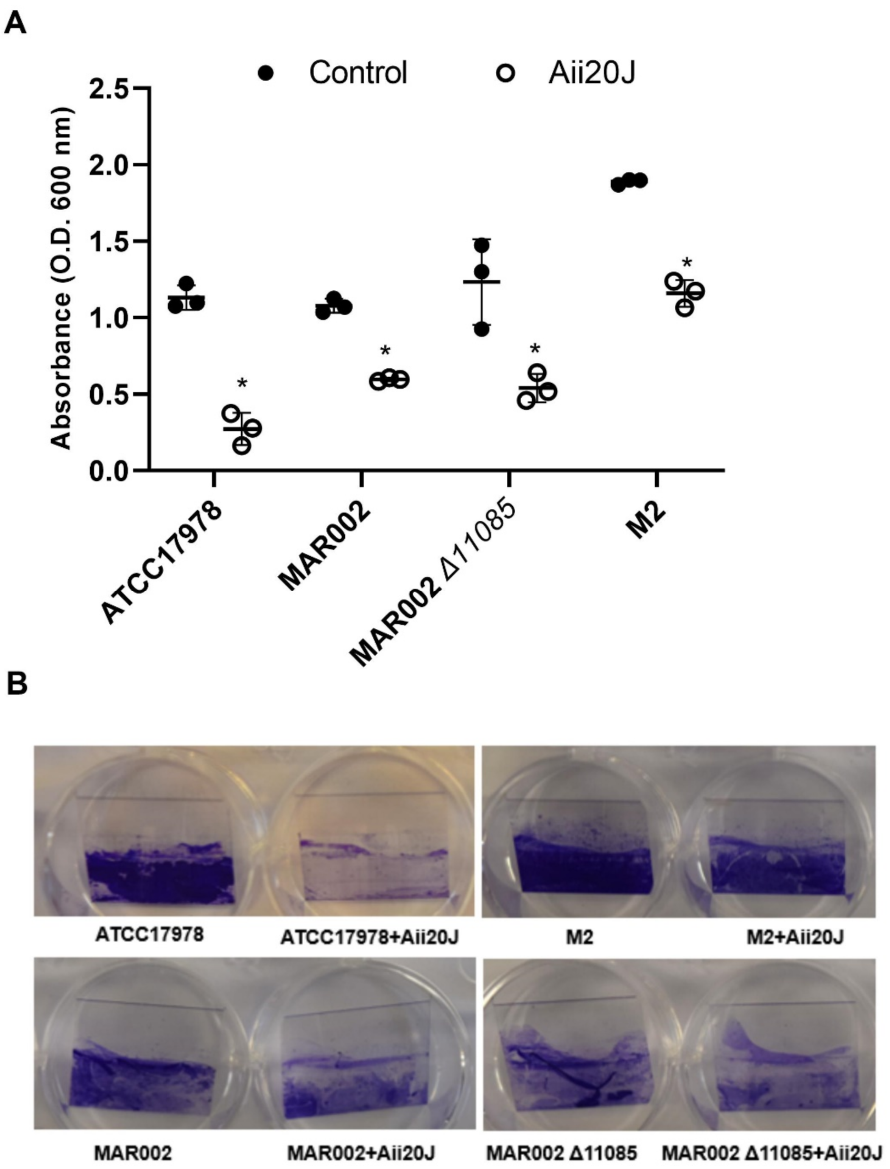


**Supplementary Figure 5.** Effect of the QQ lactonase Aii20J (20 µg/mL) on biofilm formation by *A. baumannii* ATCC® 17978™, the *A. baumannii* clinical strain MAR002, which was reported to be a biofilm hyper-former, its Δ11085 mutant, which was reported to be defective in biofilm formation and eukaryotic cell attachment in comparison with the parental strain (Álvarez-Fraga et al. 2016) and *A. nosocomialis* M2. (A) Biofilm quantification using the Crystal Violet staining method. Values are averages ±sd (n=3). The asterisks show statistically significant differences (Mann-Whitney test, $P<0.05$) between treated and untreated biofilms for the same strain. (B) pictures showing the aspect of the untreated (left) and treated (right) biofilms stained with crystal violet. Biofilms were produced on 18x18 mm glass coverslips using a modification of the *Active Attachment* model (Exterkate et al. 2010) in LS-LB, incubated for 24 h at 37ºC.
